# Dual-gene detection in a single-tube system based on CRISPR-Cas12a/Cas13a for severe fever thrombocytopenia syndrome virus

**DOI:** 10.3389/fmicb.2022.977382

**Published:** 2022-11-03

**Authors:** Yating Zhu, Chen Xing, Li Yang, Qian Li, Xiaofeng Wang, Jing Zhou, Cong Zhang, Cuiping Ren, Fahu Liu, Jun He, Bing Shen, Yinan Du, Yan Liu

**Affiliations:** ^1^School of Basic Medical Sciences, Anhui Medical University, Hefei, China; ^2^Anhui Provincial Laboratory of Microbiology and Parasitology, Department of Microbiology and Parasitology, Anhui Medical University, Hefei, China; ^3^Department of Clinical Laboratory, The Fourth Affiliated Hospital of Anhui Medical University, Hefei, China; ^4^Department of Clinical Laboratory, The Affiliated Yijishan Hospital of Wannan Medical College, Wuhu, China; ^5^Microbiological Laboratory, Anhui Center for Disease Control and Prevention, Hefei, China

**Keywords:** SFTSV, CRISPR/Cas12a, CRISPR/Cas13a, multiplex detection, point-of-care testing

## Abstract

Severe fever with thrombocytopenia syndrome (SFTS) is an emerging infectious disease, which is caused by severe fever with thrombocytopenia syndrome virus (SFTSV). The disease results in high mortality and increased morbidity and threatens global public health. Rapid detection of SFTSV is crucial for epidemic prevention in low-resource settings. Here we developed deployable, sensitive and rapid detection methods based on CRISPR/Cas12a or Cas13a technologies. The CRISPR/Cas12a-based detection assay could stably detect the SFTSV L or M genes at 10 cp/μl. The Cas13a-based method could detect the L gene as low as 0.75 cp/μl. For point-of-care testing, we combined fluorescence visualization and lateral flow detection with CRISPR/Cas-based assays. Furthermore, using the orthogonal DNA/RNA collateral activity of the Cas12a/Cas13a system, we present the dual-gene detection platform for SFTSV, which can simultaneously detect the L and M genes in a single tube. Based on the dual-gene detection, we designed multiplexed test strips to detect SFTSV. All our methods were initially validated using 52 clinical samples, showing 100% sensitivity and specificity. These new CRISPR/Cas-based detection methods are promising candidates for on-site detection of SFTSV.

## Introduction

Severe fever with thrombocytopenia syndrome (SFTS) has been recognized as a new tick-borne zoonosis in recent years. When SFTS was initially identified, the mortality rate was as high as 30% (Sun et al., 2017). SFTS is caused by severe fever with thrombocytopenia syndrome virus (SFTSV), which is in the family *Bunyaviridae*, genus *Phlebovirus*. The viral genome is made up of three segments, designated large (L), medium (M), and small (S). The L segment consists of 6,368 nucleotides, while the M segment consists of 3,378 nucleotides. Both the M and L segments are single-stranded negative RNAs that encode glycoproteins (Gn and Gc) and RNA-dependent RNA polymerase (RdRp), respectively. The S segment is ambisense RNA and contains 1744 nucleotides that mainly encode the nucleoprotein (NP) and nonstructural proteins (NSs; Zhan et al., 2017). It has been well documented that all three segments could be used for nucleic acid detection, with no clear difference in specificity or sensitivity among them (Lee et al., 2020).

Since SFTSV was isolated and identified in 2009, the virus has rapidly spread to many countries (Yu et al., 2011). A total of 5,360 cases of laboratory-confirmed SFTSV infection were reported in China between 2011 and 2016. And 172 cases were reported in South Korea from 2012 to 2015, with a mortality rate of 32.6% (Sun et al., 2017; Lee et al., 2020). In the aftermath, the SFTS cases were also reported in Japan, Vietnam, and the United Arab Emirates (Denic et al., 2011; Takahashi et al., 2014; Tran et al., 2019). Additionally, outbreaks of person-to-person transmission have occurred in endemic areas of SFTSV (Tang et al., 2013; Wang et al., 2014; Jiang et al., 2015; Huang et al., 2017). The epidemic disease caused by SFTSV poses a potential threat to global health security.

The main clinical presentations of SFTS include high fever, thrombocytopenia, leukocytopenia, gastrointestinal hemorrhage and multiple organ failure (Yu et al., 2011). Because the clinical manifestations of SFTS lack of specificity, it is difficult to distinguish SFTS from other common diseases. Also, there is currently no available vaccine or drugs for SFTS. Hence, screening the suspected SFTS cases during the early stage of infection is one of the crucial strategies for controlling the outbreak. Virus isolation is the most convincing evidence for the diagnosis of SFTSV infection (Goldsmith et al., 2013). However, this method usually takes more than 1 week to isolate and culture the virus; it is not recommended for early clinical diagnosis. And because serological antibody detection is time-consuming and labor-intensive, it is often used to monitor subacute cases (Jiao et al., 2012). At present, the most focused molecular detection methods, such as polymerase chain reaction (PCR) and quantitative real-time polymerase chain reaction (qPCR), are not suitable for on-site detection of SFTSV, due to the requirements of sophisticated instruments and skilled personnel (Sun et al., 2012). A series of isothermal amplification techniques have also been established to detect SFTSV, including loop-mediated isothermal amplification (LAMP) and recombinase polymerase amplification (RPA; Zhou et al., 2020; Sano et al., 2021). But these amplification technologies still show limitations in sensitivity and specificity.

It has been reported that the time interval from illness onset to confirmation is correlated with the case fatality rate (Sun et al., 2016). Hence, a nucleic acid detection system that is simple, sensitive and can effectively speed up the diagnosis and treatment of diseases is essential. Recently, the cluster regularly interspaced short palindromic repeats (CRISPR)/CRISPR-associated protein (Cas) technologies have brought a revolutionary improvement for molecular diagnostics. The class 2 CRISPR/Cas systems have been developed as nucleic acid detection tools with remarkable versatility and flexibility (Gootenberg et al., 2017; Chen et al., 2018). As is widely known, CRISPR/Cas9 has been broadly utilized for genome editing (Jinek et al., 2012; Cong et al., 2013). To date, considerable studies have shown the collateral activity of Cas12 or Cas13 that can nonspecifically cause non-specifically cleavage of single-stranded DNA or RNA once specifically bound to the target sequences *via* engineered guide RNAs. Based on this, newly developed nucleic acid detection technologies utilizing the CRISPR/Cas12 or Cas13 systems and combined with isothermal amplification show high sensitivity and specificity. The Cas12-based and Cas13-based detection methods have significant advantages and have been applied for the detection and genotyping of many pathogens, including Dengue virus (DENV), Zika virus (ZIKV), human papillomavirus (HPV), and African swine virus (ASFV; Gootenberg et al., 2017; Chen et al., 2018; Wang et al., 2020).

In this study, we developed the Cas12-based and Cas13-based detection assays for SFTSV. The detection can be combined with fluorescence visualization and immunochromatographic strips, which is suitable for the on-site situation. Notably, we established a rapid dual-gene detection system based on CRISPR/Cas12 and Cas13, which can simultaneously target the L and M genes of SFTSV in a single tube. We further developed the dual-channel test strips that take advantage of the high specificity and accuracy of dual-gene detection technology and the portability and practical simplicity of lateral flow detection.

## Materials and methods

### Clinical samples and RNA extraction

The clinical samples in this study were collected from The Affiliated Yijishan Hospital of Wannan Medical College. The positive specimens were collected in the acute phase of SFTSV patients. All blood samples were collected and processed under the standard operation recommended by the WHO. The blood samples were centrifuged to separate the serum. Before RNA extraction, all serum samples were inactivated by heating at 56°C for 30 min. The genomic RNA extraction was used RNeasy® Mini Kit (QIAGEN, cat. no. 74106), according to the manufacturer’s directions. All the RNA extracts were partitioned and stored at −80°C for further detection.

Ethical approval was obtained from the Biomedical Ethics Committee of Anhui Medical University (AHMU-SBMS-20172005; AHMU-SBMS-20210742).

### Nucleic acid and crRNAs preparation

The primers and fluorescent reporters were synthesized by TsingKe (Nanjing, China). The sequences are listed in the Supplementary Table.

The target fragments of the L gene and M gene from the HB29 strain were cloned into the pMD19-T vector, respectively. The SFTSV RNA standards were transcribed with the vectors, using HiScribe™ T7 Quick High Yield RNA Synthesis Kit (NEB, cat. no. E2050S) based on the manufacturer’s directions. After transcription, the RNA products were treated with DNase I to remove template DNA and then purified using Monarch® RNA Cleanup Kit (NEB, cat. no. T2040L). The concentration of RNA standards was measured using the Nanodrop Spectrophotometer (ND-2000), and the purity and integrity of RNA were determined by electrophoresis. The RNA standards were aliquoted and stored at-80°C. The sequences are detailed in the Supplementary Table.

The oligonucleotides complementary to designed crRNAs were synthesized by TsingKe (Nanjing, China). For *in vitro* transcription of crRNAs, the complementary sequence of the T7 promoter must be appended to the 3′ end of the synthetic DNA oligonucleotides. The transcription and purification of crRNAs were similar to that previously described for SFTSV RNA standards. The sequences of all crRNAs are detailed in the Supplementary Table.

### Isothermal amplification

The isothermal recombinase-aid amplification of SFTSV was performed using a commercial RT-RAA kit (ZhongCe Bio-Sci& Tech Co., Ltd., Hangzhou, China) following the manufacturer’s directions. Lyophilized pellets per tube were rehydrated with a 50 μl reaction mixture. The 50 μl reaction mixture contained 41.5 μl rehydrated buffer (A buffer), 2 μl forward primer (10 μM), 2 μl reverse primer (10 μM), and 2 μl RNA template. Next, 2 μl magnesium acetate (B buffer) was added to the lid of each reaction tube, and the lid was closed carefully. After thoroughly mixing and brief centrifugation, the reaction tube was incubated at 37°C or 42°C for 20 min; in the version of dual-gene detection for SFTSV, 1 μl of each forward primer (10 μM) and 1 μl of each reverse primer (10 μM).

### CRISPR/Cas12a detection assay

The 1 μl LbaCas12a (1 μM; NEB, cat. no. M0653S), 1 μl Cas12a-crRNA (1 μM), and 0.5 μl RNase inhibitor, Murine (40 U/μl, NEB, cat. no. M0314L) were preincubated with 2 μl NEB buffer 2.1 (1×) in the volume of 20 μl at 37°C for 10 min. After forming the RNA-protein (RNP) complexes, the 0.1 μl ssDNA reporter (100 μM, FAM-BHQ) was added to the reaction mixture. The reaction mixture was gently mixed and immediately placed on ice until use. Then, the CRISPR/Cas12a detection assay was performed with 2 μl amplification products and 18 μl of the reaction mixture at 37°C for 20 min. The Bio-Rad CFX96 Real-Time PCR System (Bio-Rad, USA) was used to monitor the fluorescence and the fluorescence images were acquired every 2 min.

### CRISPR/Cas13a detection assay

The 0.4 μl HEPES (1 M; Gibco, cat. no. 15630–106), 0.2 μl MgCl_2_ (1 M; Invitrogen, cat. no. AM9530G), 2 μl Ribonucleotide Solution Mix (25 mM; NEB, cat. no. N0466L), 2 μl LwaCas13a (1 μM; Genscript), 1 μl Cas13a-crRNA (1 μM), 0.1 μl T7 RNA polymerase (50 U/μl, Lucigen, cat. no. 30223–1), 1 μl Murine RNase inhibitor (40 U/μl; NEB, cat. no. M0314L), and 0.2 μl ssRNA reporter (100 μM, FAM-BHQ) were added to 20 μl reaction mixture. The reaction mixture was gently mixed and immediately placed on ice until use. Next, a 2 μl amplification product was added to an 18 μl reaction mixture for CRISPR/Cas13a detection assay. The detection assay was performed at 37°C for 20 min, while the fluorescence was monitored as described above. The fluorescence images were acquired every 2.5 min.

### Dual-gene detection assay

The 1 μl LbaCas12a (1 μM; NEB, cat. no. M0653S), 1 μl Cas12a-crRNA (1 μM), 1.6 μl NEB buffer 2.1 (1×), and 10.4 μl RNase-free ddH_2_O were preincubated at 37°C for 10 min. After fully formation of RNA-protein complex, 0.4 μl HEPES (1 M; Gibco, cat. no. 15630–106), 0.2 μl MgCl_2_ (1 M; Invitrogen, cat. no. AM9530G), 0.2 μl NaCl (5 M; Invitrogen, cat. no. AM9760G), 0.8 μl Ribonucleotide Solution Mix (25 mM; NEB, cat. no. N0466L), 2 μl LwaCas13a (1 μM; Genscript), 1 μl Cas13a-crRNA (1 μM), 0.1 μl T7 RNA polymerase (50 U/μl, Lucigen, cat. no. 30223-1), 1 μl Murine RNase inhibitor (40 U/μl; NEB, cat. no. M0314L), 0.1 μl ssDNA reporter (100 μM, VIC-BHQ), and 0.2 μl ssRNA reporter (100 μM, FAM-BHQ) were mixed with the preincubated system in a final volume of 20 μl reaction mixture. The 2 μl amplification product was added into an 18 μl reaction mixture for the dual-gene detection. The reaction tube was incubated at 37°C for 20 min. The fluorescent intensity was monitored in the FAM and VIC channels simultaneously and the fluorescence images were acquired every 2.5 min. The procedure of optimizing conditions mainly included adjusting the priority of Cas12a and crRNA pre-incubation and adjusting the concentration of rNTPs.

### The lateral flow detection

In the lateral flow detection of the single-target test strip, the ssDNA reporter or ssRNA reporter was labeled with FAM and biotin. Moreover, for the lateral flow detection of the multiplexed test strip, the ssDNA reporter was labeled with digoxin and biotin, and the ssRNA reporter was labeled with FITC and biotin. After the Cas enzymes mediated trans-cleavage assay, the CRISPR detection system was diluted 1:3 in the detection buffer. Then, we inserted immunochromatographic strips (Novel gene, Hefei, China) or dropped the dilution onto the strips and incubated for 5 min at room temperature. Next, the detection results of the testing strips were visually inspected and photographed.

### Quantitative real-time PCR detection

We used Luna® Universal Probe One-Step RT-qPCR Kit (NEB, cat. no. E3006L) to perform the dual-channel qPCR detection (detecting the L gene in Hex channel and M gene in FAM channel) of SFTSV following the procedure recommended by the Chinese Center for Disease Control and Prevention (China CDC). The single-tube qPCR Mix was prepared to contain 10 μl Luna® Universal Probe One-Step Reaction Mix (2×), 1 μl Luna® WarmStart® RT Enzyme Mix (20×), 0.4 μl L-probe, 0.4 μl M-probe, 0.4 μl L forward primer, 0.4 μl L reverse primer, 0.4 μl M forward primer, 0.4 μl M reverse primer, 2 μl RNA extracts, and 5.4 μl RNase-free ddH_2_O. The detection assay was carried out using the Bio-Rad CFX96 system with the following program: reverse transcription at 55°C for 10 min, pre-denaturation at 95°C for 1 min, and followed by 45 cycles of denaturation at 95°C for 10 s, annealing and extension at 60°C for 30 s. The specimens with cycle threshold (Ct) values ≤38.0 were considered SFTSV positive.

## Results

### Establishment of detection method for SFTSV based on CRISPR/Cas12a

**Figure 1:**
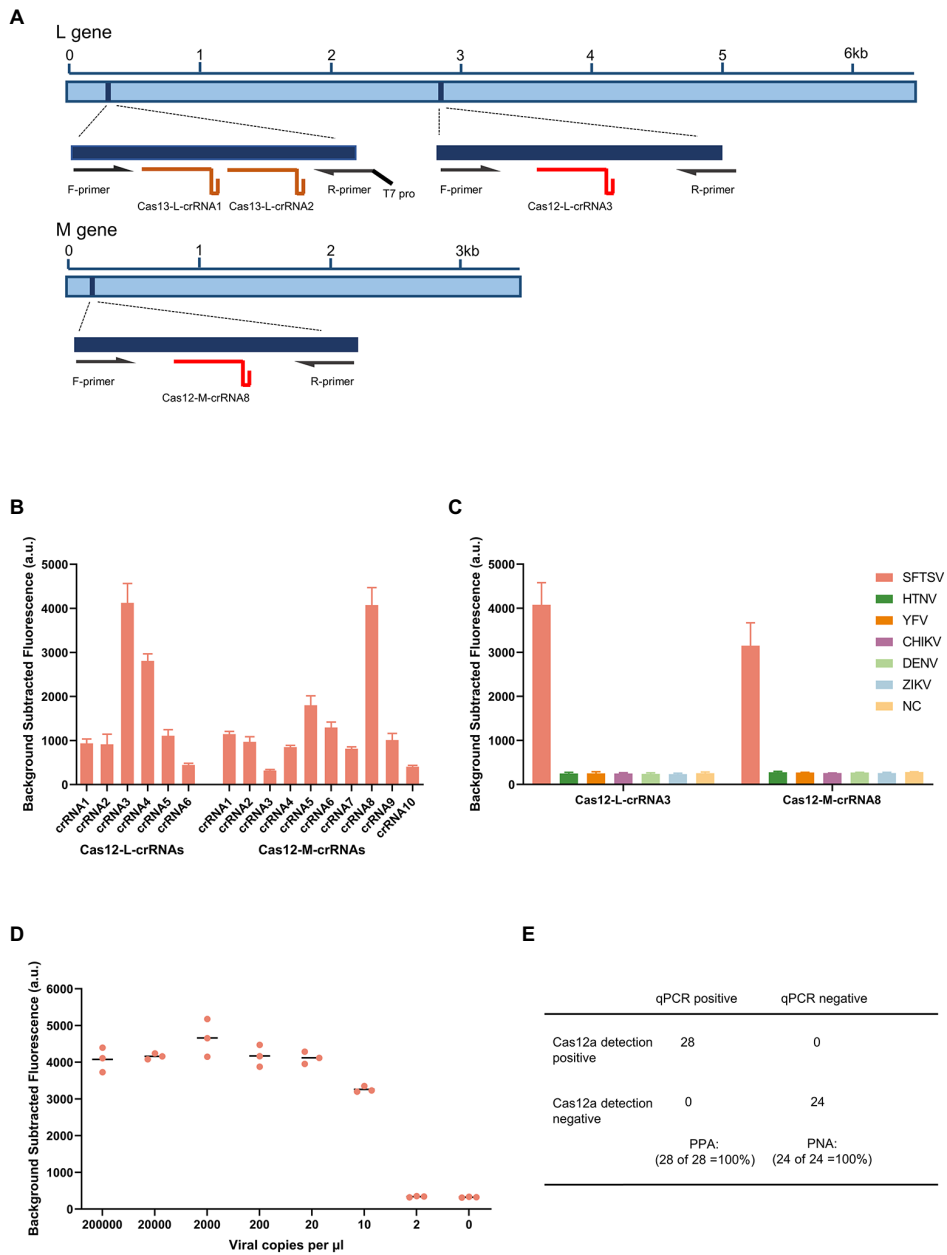
Establishment of detection method for SFTSV based on CRISPR/Cas12a. **(A)** Schematic diagram of the L gene and M gene of SFTSV. The gene map shows the location of selected regions, corresponding primers and crRNAs. T7 pro, T7 polymerase promoter. **(B)** The designed LbaCas12a-crRNAs detect the targets of L gene and M gene that were amplified using the respective primer mix. The Cas12-L-crRNA3 and Cas12-M-crRNA8 showed stronger signals using the same protocol. Values represent mean ± SD (*n* = 3). **(C)** SFTSV, Hantaan virus, Yellow fever virus, Chikungunya virus, Dengue virus, and Zika virus were detected parallelly by Cas12-L-crRNA3 or Cas12-M-crRNA8. Only the RNA from SFTSV produced detected signals, whereas RNAs from other viruses and the negative control did not produce any change of signals. NC, no-template control. Values represent mean ± SD (*n* = 3). **(D)** The limit of detection for Cas12a-based assay using Cas12-M-crRNA8. The diluted SFTSV RNA standards were used as detection targets (*n* = 3). **(E)** Comparison of the clinical performance of Cas12a-based assay and qPCR assay. A total of 52 clinical samples (28 positives and 24 negatives) were evaluated using the fluorescent version of CRISPR/Cas12a assay and qPCR assay; PPA, percent positive agreement. PNA, percent negative agreement.

To detect SFTSV, we selected the L and M gene’s highly conserved regions as the target fragments (Figure 1A). Following the principle that LbaCas12a recognized the target sequence with a thymine (T) nucleotide-rich protospacer adjacent motif, we designed different Cas12a-crRNAs to detect the L gene and M gene, respectively (Supplementary Table). We firstly screened out Cas12-L-crRNA3 and Cas12-M-crRNA8 for subsequent experiments, which performed best among the detection of standards (Figure 1B). Since the oligonucleotide sequence greatly impacts on the efficiency of isothermal amplification, and the amplification efficiency would further determine the detection sensitivity, we designed at least three pairs of RT-RAA primers for each amplicon (Supplementary Table). Then, we evaluated diverse primer combinations for each target to develop a preferred detection method. To filter out the optimal primer pairs, we used a reverse primer (R1) against different forward primers (F1 to F3), and the best forward primer (F2) was selected. Next, F2 was used to screen all reverse primers (R1 to R3). Ultimately, the primer combination (F2 + R1) of Cas12-L-crRNA3 with the best performance was selected for further research (Supplementary Figures 1A,B). The same procedures were also performed to optimize of primer pairs of Cas12-M-crRNA8 (Supplementary Figures 1C,D).

To access the specificity of the detection method based on CRISPR/Cas12a, we tested against the RNA extracted from other related viruses using Cas12-L-crRNA3 and Cas12-M-crRNA8, respectively. These related viruses are common hemorrhagic fever viruses that cause similar clinical symptoms to SFTSV. No cross-reaction was observed in the detection (Figure 1C). Next, we analyzed the sensitivity of the detection assay based on the Cas12a system for SFTSV. The serially diluted RNA standards were used as templates, and we multiplied the detectable nucleic acids by isothermal amplification, before the Cas enzyme detection step. In addition, we detected SFTSV RNA standards using the double-channel qPCR assay in parallel as a reference, since it was recognized as the gold standard for viral nucleic acid testing (Supplementary Figure 2). All experiments were conducted under double-blind conditions to eliminate subjective factors that could impact the results. The experimental results illustrated that the detection assay based on CRISPR/Cas12a could stably detect the M gene using Cas12-M-crRNA8 or the L gene using Cas12-L-crRNA3 at 10 cp/μl (Figure 1D; Supplementary Figure 3A). To verify the clinical sensitivity of the method, we used a total of 52 clinical samples for SFTSV detection (Supplementary Figure 4). Finally, the overall concordance of positive/negative detection results for SFTSV showed 100% between the CRISPR/Cas12a assay and qPCR (Figure 1E).

### The ultrasensitive detection assay based on CRISPR/Cas13a

**Figure 2:**
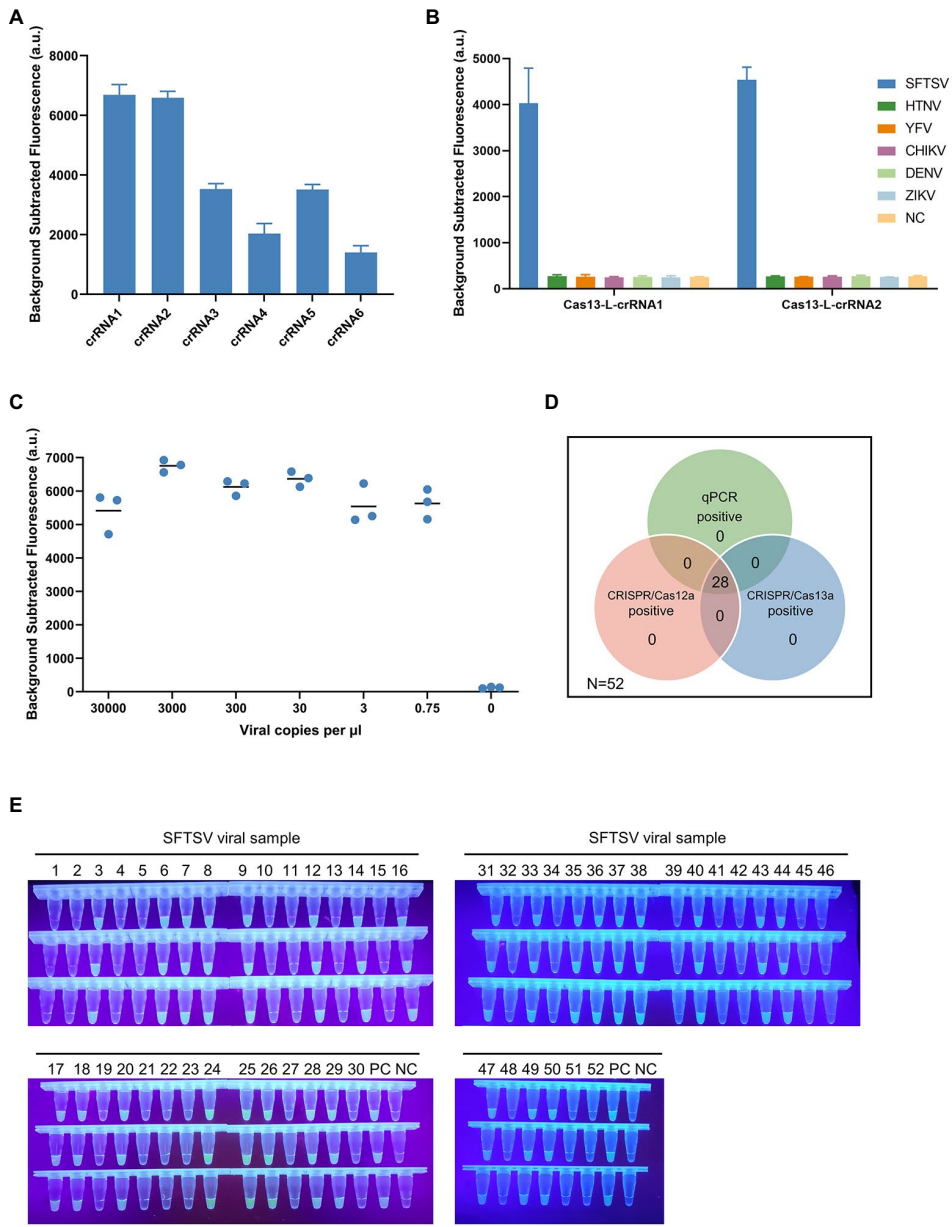
The ultrasensitive detection assay based on CRISPR/Cas13a. **(A)** The designed LwaCas13a-crRNAs were used to detect the targets of L gene that was amplified by primer mix. The Cas13-L-crRNA1 and Cas13-L-crRNA2 exhibited stronger signals using the same protocol. Values represent mean ± SD (*n* = 3). **(B)** The specificity of Cas13-L-crRNA1 and Cas13-L-crRNA2. Several hemorrhagic fever viral RNA was detected parallelly using Cas13-L-crRNA1 or Cas13-L-crRNA2. The experimental results showed that there was no cross-reactivity in the detection. Values represent mean ± SD (*n* = 3). **(C)** The sensitivity of Cas13a-based assay using Cas13-L-crRNA1. The diluted SFTSV RNA standards were used as detection targets. (*n* = 3). **(D)** The Venn diagram showed the concordance of the results of the three assay systems on clinical samples. **(E)** The fluorescence visualization of the Cas13a-based assay for detection of clinical samples. A total of 52 samples were used for the evaluation. The results were observed and took pictures at 20 min for the Cas13a reaction under ultraviolet light (*n* = 3). PC, positive control; NC, no-template control.

There have been reports on viral nucleic acid detection using the CRISPR/Cas13a system which achieves higher sensitivity. We next combined isothermal amplification with CRISPR/Cas13a technology to establish a sensitive detection method for SFTSV. Firstly, we designed six specific LwaCas13a-crRNAs to detect the L gene of SFTSV. We then selected Cas13-L-crRNA1 and Cas13-L-crRNA2 as the targets for further study, according to preliminary screening results (Figure 2A). Subsequently, with various primer pairs for isothermal amplification subjected to the screening procedure, we identified the optimal primer combinations (Supplementary Figure 5). To determine the specificity of the CRISPR/Cas13a-based assay for SFTSV, we used Cas13-L-crRNA1 and Cas13-L-crRNA2 to test the extracts of the related viruses (Figure 2B). The intensity of the fluorescence signal showed that the Cas13a method only targeted the L gene of SFTSV, with no cross-reactivity. Then, we explored the limit of detection for the Cas13a assay. Using serially diluted SFTSV RNA standards, we found that the sensitivity of the CRISPR/Cas13a-based assay is much better than the detection based on CRISPR/Cas12a. We observed that the assay using Cas13-L-crRNA1 could detect the target of the L gene at 0.75 cp/μl, while the assay using Cas13-L-crRNA2 could detect at 3 cp/μl (Figure 2C; Supplementary Figure 3B). The performance of this CRISPR/Cas13a-based assay was also validated by testing the extracts from the 52 clinical samples (Supplementary Figure 6). As we might expect, the results of SFTSV detection showed 100% consistency among the three detection methods for both positive and negative samples (Figure 2D).

To make the detection results easily accessible, we combined the fluorescence visualization after the Cas-mediated probe cleavage with the field detection system. The positive detections could emit green fluorescence visible to the naked eye readout under ultraviolet light, while the negative detections were transparent. And the strategy of fluorescence visualization can be applied to clinical samples, and the detection results verified its diagnostic accuracy in the situation without a fluorescence reader (Figure 2E; Supplementary Figure 7).

### Development of dual-gene detection system for SFTSV

**Figure 3:**
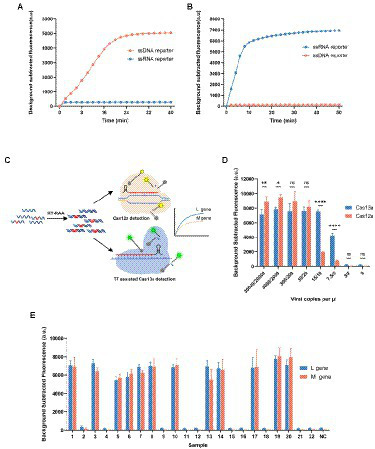
Development of dual-gene detection system for SFTSV. **(A, B)** Both ssDNA reporter (FAM-BHQ) and ssRNA reporter (VIC-BHQ) were added in the detection of Cas12a or Cas13a, and observed the changes in the two kinds of fluorescence intensity. Only the ssDNA reporter was cleaved by the activated Cas12a, and only the ssRNA reporter was cleaved by the activated Cas13a. **(C)** The schematic diagram of the dual-gene detection system for SFTSV with LbaCas12a and LwaCas13a. The L gene was detected in the FAM channel and M gene in the VIC channel. **(D)** Determination of the sensitivity of the multiplexed detection system using synthetic RNA standards after the condition optimization. The detectable signal at 10 cp/μl for Cas12a channel was weaker and unstable. Values represent mean ± SD (*n* = 3). Statistical significance for comparison was determined using Two-way ANOVA and significance was considered as ns ≥ 0.05, *p < 0.05, **p < 0.01, ****p < 0.0001. **(E)** The results of SFTSV detection in 22 clinical samples (12 positives and 10 negatives) using the multiplexed detection system. The L gene was detected by Cas13a using Cas13-L-crRNA1 and the M gene was detected by Cas12-M-crRNA8. Values represent mean ± SD (*n* = 3).

We have proved that both Cas12a and Cas13a could be used to rapidly detect SFTSV; each method still has several limitations that can be further improved. For example, each detection method can only detect a single target in one reaction, because the collateral activity of Cas12a or Cas13a is non-specific. But many nucleic acid testing applications currently require the simultaneous detection of multiple targets to ensure the stability of the results. Therefore, we sought to develop a multiplex detection system based on CRISPR that can identify different targets in a single reaction. It is well known that Cas enzymes from different species have different cleavage sequence preferences. And the Cas12a can only non-specifically cleavage ssDNA, while Cas13a can only non-specifically cleavage ssRNA. Thus, the multiplex detection approach can utilize the different cleavage preferences of Cas12a and Cas13a to discriminate the targets. Motivated by this, we developed the dual-gene detection system for SFTSV, which detected the L gene in the Cas13a channel and the M gene in the Cas12a channel (Figure 3C).

It has been reported that the trans-activated LbaCas12a cuts the ssDNA reporter, and LwaCas13a cuts the ssRNA reporter. To explore whether the two detection systems are compatible, we verified the orthogonality of the collateral activity of Cas12a and Cas13a. To determine the characteristics of cleavable substrates in Cas-mediated cleavage, we added simultaneously ssDNA and ssRNA reporters in the reaction of Cas12a or Cas13a (Figures 3A,B). We observed that the two Cas enzymes are activated and only cleavage their preferred homopolymer reporter, respectively. It is indicated that the two Cas enzymes have orthogonal collateral activity, without mutual interference and cross-reactivity.

Because the optimum temperature interval of LbaCas12a and LwaCas13a are compatible, we simply incorporated the two reaction systems into one tube to couple the activity of the two Cas enzymes. But the preliminary evaluation showed that the detection efficiency of Cas12a in multiplex detection reaction is slightly reduced compared with that in single-channel reaction, especially when detecting low concentrations of targets (Supplementary Figure 8). We then focused on screening the factors that affected the cleavage and optimizing conditions to make the detectable signal of the reaction system more sensitive and robust. After a thorough screening, we found the pre-incubation of Cas12a protein with crRNA was a key factor. The Cas12a protein and Cas12a-crRNA should be incubated at 37°C for 10 min to form the Cas12a-crRNA binary complex before adding Cas13a components. We speculated that Cas13a added to the reaction system prematurely might affect the formation of the Cas12 RNP complexes. Furthermore, the components of the Cas13a detection system in the reaction are considered abundant and various; we explored the effect of the composition of the buffer on reaction efficiency. And then we have effectively improved the efficiency of the Cas12a assay by reducing the concentration of rNTPs (Supplementary Figure 8).

We further evaluated the sensitivity of the optimized dual-gene detection system for SFTSV using the serially diluted RNA standards. The minimal detection level in the Cas13a channel for the L gene was 7.5 cp/μl and in the Cas12a channel for the M gene was 20 cp/μl (Figure 3D). To clinically validate the optimized dual-gene detection system as a new detection method for SFTSV, 22 clinical samples were drawn for testing from the 52 samples previously detected by qPCR. Compared with the qPCR assay, the dual-gene detection assay correctly identified the 12 positive and 10 negative samples (Figure 3E). The results demonstrated virtually no difference between the CRISPR/Cas-based dual-gene detection system and qPCR in the clinical test for SFTSV.

### SFTSV nucleic acid detection combined with immunochromatography

**Figure 4:**
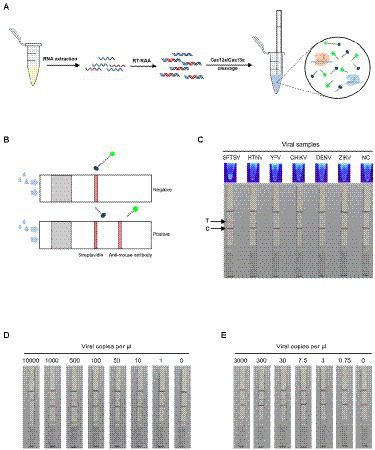
SFTSV nucleic acid detection combined with immunochromatography. **(A)** Workflow for the SFTSV RNA detection in clinical samples using CRISPR/Cas12a or Cas13a in combination with immunochromatographic strips. **(B)** Schematic of the fluorescent version of Cas12a/Cas13a-based assay coupled with lateral flow detection. The intact ssDNA/ssRNA reporter labeled FAM and biotin flows to the control capture line. When the ssDNA/ssRNA reporter was cleaved by activated Cas12a/Cas13a, the cleaved reporter flows to the target capture line. **(C)** The specificity of fluorescence visualization and lateral flow detection based on CRISPR/Cas12a assay for SFTSV. The top line is the test line, and the bottom line is the control line. The color change at the test line was only observed for SFTSV detection. **(D)** The detection limit of the single-target test strip based on CRISPR/Cas12a assay using Cas12-M-crRNA8. **(E)** The detection limit of the single-target test strip based on CRISPR/Cas13a assay using Cas13-L-crRNA1.

An easy-to-visualize readout mode is crucial for field-deployable detection of SFTSV. In addition to fluorescence visualization, we combined the immunochromatography lateral flow strips with a CRISPR/Cas12a or Cas13a detection assay (Figure 4A). We introduced FAM-biotin reporter into Cas12a or Cas13a cleavage for single-target detection. The undivided reporter would only be captured at the control line on the lateral flow strips. When the target was present, the reporter would be cleaved by the Cas enzyme, then the cleaved FAM-labelled reporter could flow through the control line and be captured at the test line. On the test line, the rabbit anti-mouse antibody could bind the cleaved reporter with the mouse anti-FAM antibody conjugated to gold nanoparticles (Figure 4B). As shown in Figure 4C, the lateral flow single-target detection based on Cas12a showed adequate specificity, consistent with fluorescence signals. With the serially diluted SFTSV RNA standards, we demonstrated that the test strips combined with Cas12a assay could detect the M gene at 10 cp/μl, while combined with Cas13a assay can detect the L gene at 3 cp/μl (Figures 4D,E). Concomitantly, we tested the feasibility of the single-target test strips in clinical applications using the 52 clinical samples, which were in 100% agreement with fluorescent version results (Supplementary Figure 9).

### Multiplex targets detection of SFTSV RNA on a lateral flow strip

**Figure 5:**
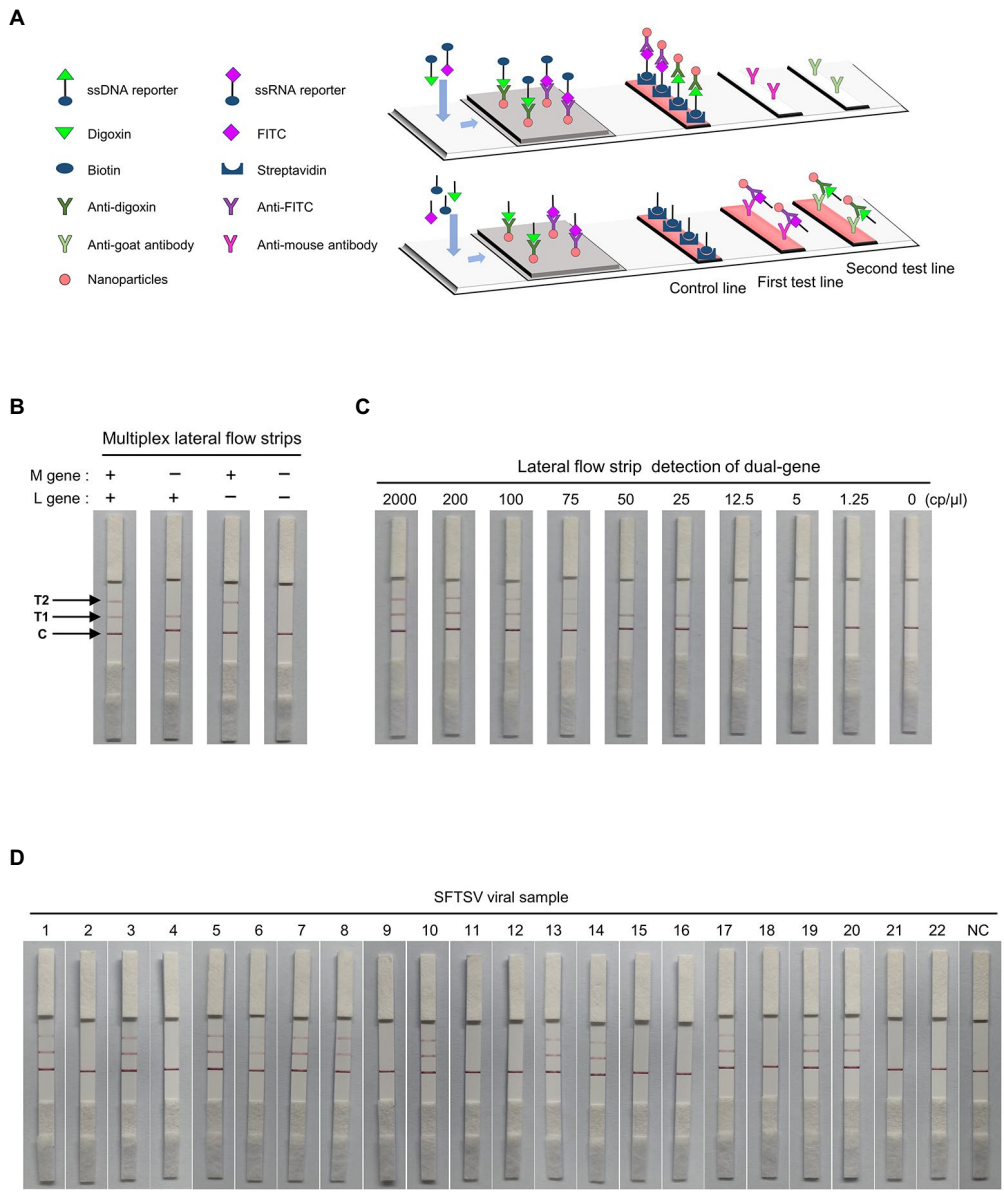
SFTSV nucleic acid detection combined with immunochromatography. **(A)** Schematic diagram of the multiplexed test strips. **(B)** The multiplexed test strip assay readout. The positive result requires detection of at least one of the two targets (L gene at the first target capture line and M gene at the second target capture line). **(C)** The sensitivity of multiplexed test strips based on the dual-gene detection system using the diluted synthetic RNA standards. **(D)** The detection results of multiplexed lateral flow strips for 22 clinical samples. NC, no-template control.

Moreover, we further applied the dual-gene detection system to the design of test strips and established multiplex detection in a single lateral flow strip for SFTSV (Figure 5A). The multiplexed test strips combined with the collateral activity of Cas12a and Cas13a simultaneously, consisting of one control line and two test lines. In the dual-gene detection system, if the L gene of SFTSV is present since the ssRNA reporter is labeled with FITC and biotin, allowing capture with an anti-mouse antibody on the first test line. Meanwhile, if the M gene is present since the ssDNA reporter is labeled with digoxin and biotin, the split reporter can be captured on the second test line with an anti-goat antibody (Figure 5B). The multiplexed test strip could detect the L gene and M gene simultaneously, avoiding the possible false-positive or false-negative results of the single-target test strip. We evaluated the limits of the multiplexed test strip using serial dilutions of SFTSV RNA standards; the strip could stably detect 25 cp/μl in the Cas13a channel for the L gene and 75 cp/μl in the Cas12a channel for the M gene (Figure 5C). Analysis of the RNA extracts of the 22 SFTSV samples selected from 52 samples previously tested by qPCR showed that the results of multiplexed test strips were concordant with the qPCR assay (Figure 5D).

## Discussion

Since 2009, SFTS has spread in today’s tightly connected world and has become an emerging public health concern (Yu et al., 2011). The patients with SFTSV share similar clinical symptoms and disease courses with hemorrhagic fever renal syndrome, increasing the difficulty of diagnosis. A prospective study has reported that people of any age group might be susceptible to SFTSV, and elderly people tend to develop severe cases and even die from SFTSV infection (Ding et al., 2014). Furthermore, clusters of cases resulting from person-to-person transmission through contact with the blood or body fluids of patients have been reported, which has posed a challenge to epidemic prevention (Tang et al., 2013; Wang et al., 2014; Jiang et al., 2015; Huang et al., 2017). Generally, the predominant cases of SFTSV infection are farmers who lived in rural areas with poor access to medical treatment services in time (Sun et al., 2017). However, PCR or qPCR requires sophisticated equipment and professional operation, and it is hard to deploy rapidly in the field (Sun et al., 2012). To address this, we developed rapid and deployable detection methods for SFTSV. Unlike the conventional nucleic acid detection methods, our CRISPR/Cas-based methods for SFTSV involve a substantially shorter operation time and avoid the need for thermal circulation apparatus. The whole detection process was easily conducted at 37°C within 1 h. Until now, the Cas12a-based detection method for SFTSV has been reported and similar work was carried out in this study (Huang et al., 2022). Moreover, we first proposed the detection method based on CRISPR/Cas13a for SFTSV, and the lowest detection limit was 0.75 cp/μl. We noted that Cas13a-based detection might be more sensitive than Cas12a-based detection in this study, potentially due to differences in the detection process or different targets. We used the fluorescence visualization and immunochromatography test strips combined with the CRISPR/Cas12a or Cas13a detection system. The fluorescence visualization and immunochromatographic strips have the same intrinsic advantages as being portable and simple, providing a good option for POCT.

Of note, we established the orthogonal Cas13a and Cas12a detection system that can rapidly detect the L and M gene of SFTSV in a single tube. We combined the dual-gene detection system with immunochromatography to develop the multiplexed test strips, which can avoid the risk of a small percentage of false positives or negatives in single-target test strips to ensure the stability of the detection results. To our knowledge, this study is the first to report the dual-gene detection in a single tube based on CRISPR/Cas for SFTSV. The multiplexed detection system exhibits some promising advantages, including diagnostic accuracy comparable to multiplex qPCR and improved testing efficiency, and the system can be optimized to detect and differentiate two or more pathogens simultaneously.

Despite the novel reports in our study, several limitations warrant discussion. Conventional RNA extraction is time-consuming, and often a bottleneck for the molecular diagnosis of viral infections. Many reports have shown that brief heating of samples in various buffers can lyse pathogens and release nucleic acids to shorten processing time (Myhrvold et al., 2018). But it is unclear whether assay sensitivity would be compromised when the sample is lysed in different recipes, which would require further investigation and optimization. In addition, our multiplexed detection assay optimization could be developed to integrate with a smartphone app to reduce artificial bias in analyzing the data (Barnes et al., 2020). Last but not least, our detection methods can be integrated with other new technology, such as microfluidic applying high-throughput testing, surface plasmon resonance (SPR) enhancing the fluorescence signals, and new Cas effectors achieving multiplexed identification of more targets in a single tube (Li et al., 2019; Bruch et al., 2021; Zheng et al., 2022).

In conclusion, we developed a novel detection method based on CRISPR/Cas, which can be applied to the portable and sensitive nucleic acid detection of SFTSV. The multiplexed detection system has great potential to be rapidly developed and deployed in the face of clinical or practical on-site detection.

## Data availability statement

The original contributions presented in the study are included in the article/Supplementary material, further inquiries can be directed to the corresponding author.

## Ethics statement

The studies involving human participants were reviewed and approved by Biomedical Ethics Committee of Anhui Medical University. The patients/participants provided their written informed consent to participate in this study. Written informed consent was obtained from the individual(s) for the publication of any potentially identifiable images or data included in this article.

## Author contributions

YZ, CX, YD, and YL conceived and designed the study. YZ, CX, QL, and XW performed experiments and analyzed the data. YZ wrote the original draft of the manuscript. LY, CZ, and CR wrote sections of the manuscript. JZ, FL, JH, and YL collected and processed the materials. BS conceived and prepared the test strips. YD and YL contributed to funding acquisition, project administration and writing-review. All authors contributed to the article and approved the submitted version.

## Funding

This work was supported by the National Key Research and Development Program of China (2021YFC2301100), the Key Research and Development Program of Anhui province (202104a07020031), and the National Natural Science Foundation of China (31701162, 81772203, and 81802027).

## Conflict of interest

The authors declare that the research was conducted in the absence of any commercial or financial relationships that could be construed as a potential conflict of interest.

## Publisher’s note

All claims expressed in this article are solely those of the authors and do not necessarily represent those of their affiliated organizations, or those of the publisher, the editors and the reviewers. Any product that may be evaluated in this article, or claim that may be made by its manufacturer, is not guaranteed or endorsed by the publisher.
