# Supplementary material for: Dual-gene detection in a single-tube system based on CRISPR-Cas12a/Cas13a for severe fever thrombocytopenia syndrome virus
Source: Front Microbiol. 2022 Nov 3;13:977382. doi: 10.3389/fmicb.2022.977382 (PMC9668895; doi:10.3389/fmicb.2022.977382)
Supplement: Supplementary file 1 [file Data_Sheet_1.docx]

Supplementary Material

## Supplementary Figures

**
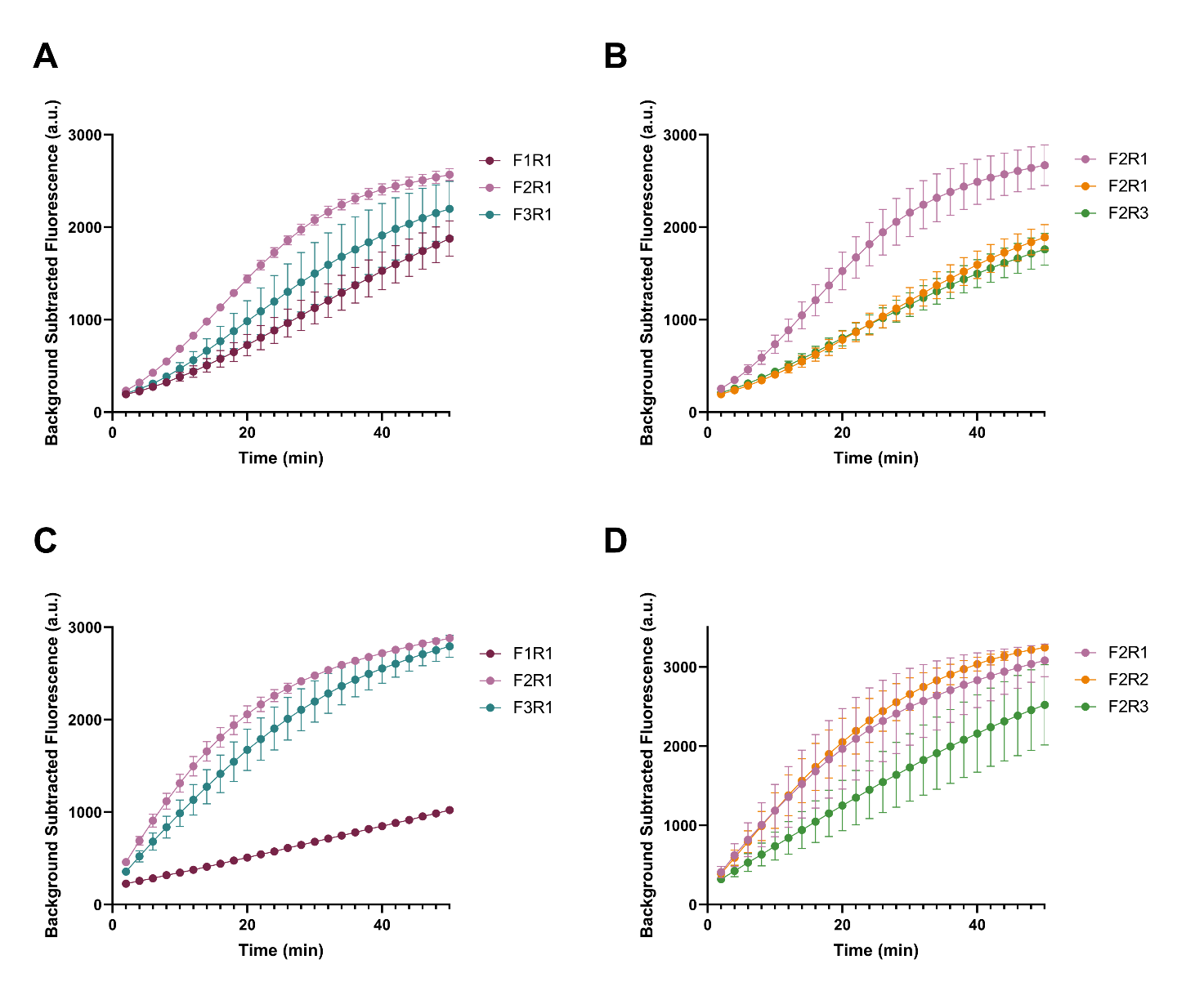
**

**Supplementary Figure 1.** Primer Screening. Primers of Cas12-L-crRNA3 (A, B) and Cas12-M-crRNA8 (C, D) were screened using a single reverse primer against the corresponding forward primers, the forward primer with the best performance was selected to perform the second round of screening. Values represent mean±SD (n=3).


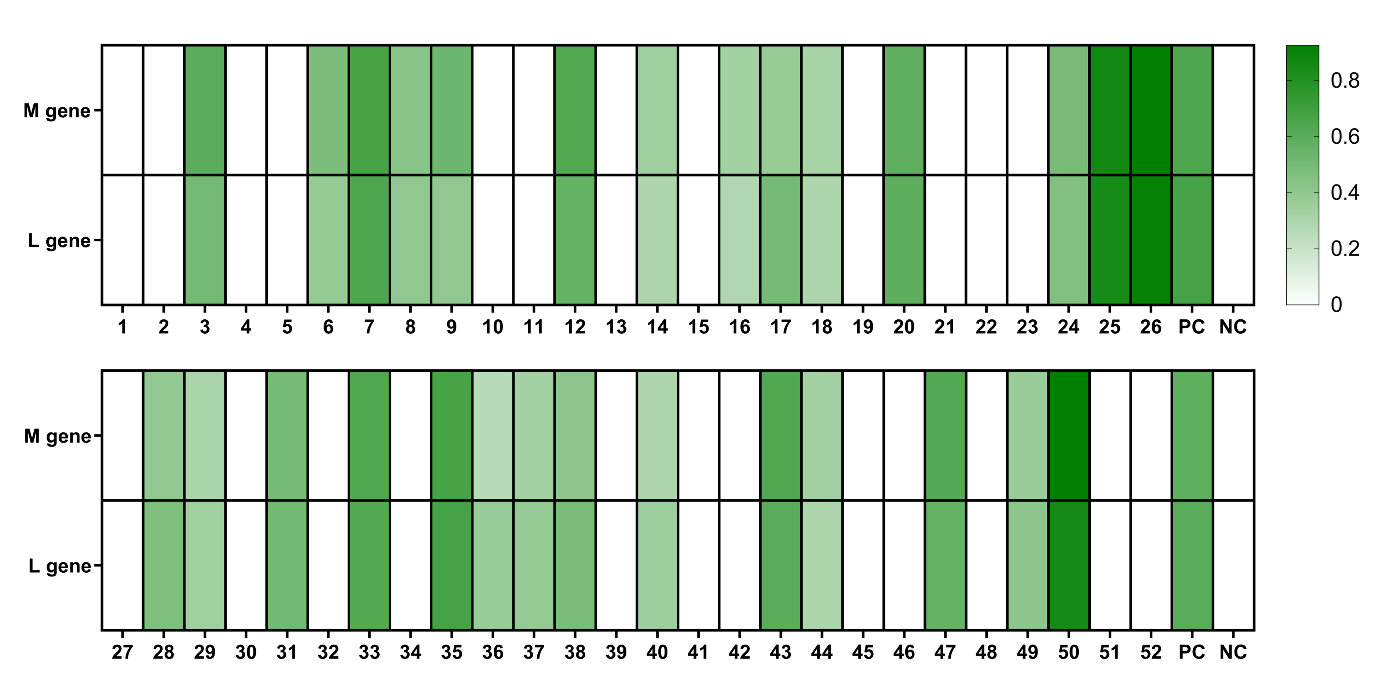


**Supplementary Figure 2.** The heatmap shows the results of dual-channel qPCR assay for SFTSV in clinical samples (n=52, three replicates). Ct values in the two channels were normalized. PC, positive control. NC, no-template control.


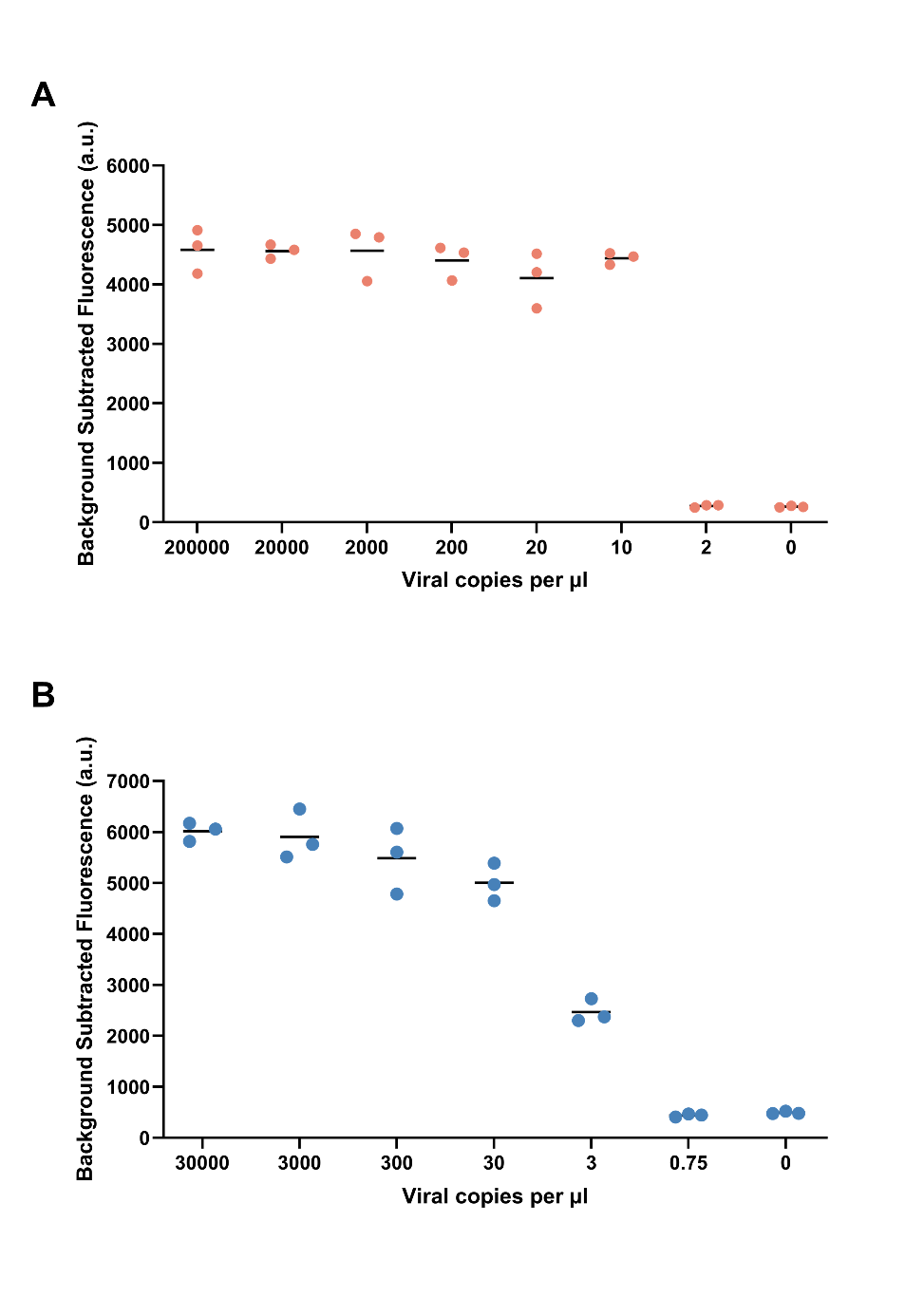


**Supplementary Figure 3.** The sensitivity of detection assay based on Cas12a or Cas13a. (A) The lowest limit of detection for L gene using Cas12-L-crRNA3. (B) The lowest of detection for L gene using Cas13-L-crRNA2. The diluted SFTSV RNA standards were used as detection targets (n=3).


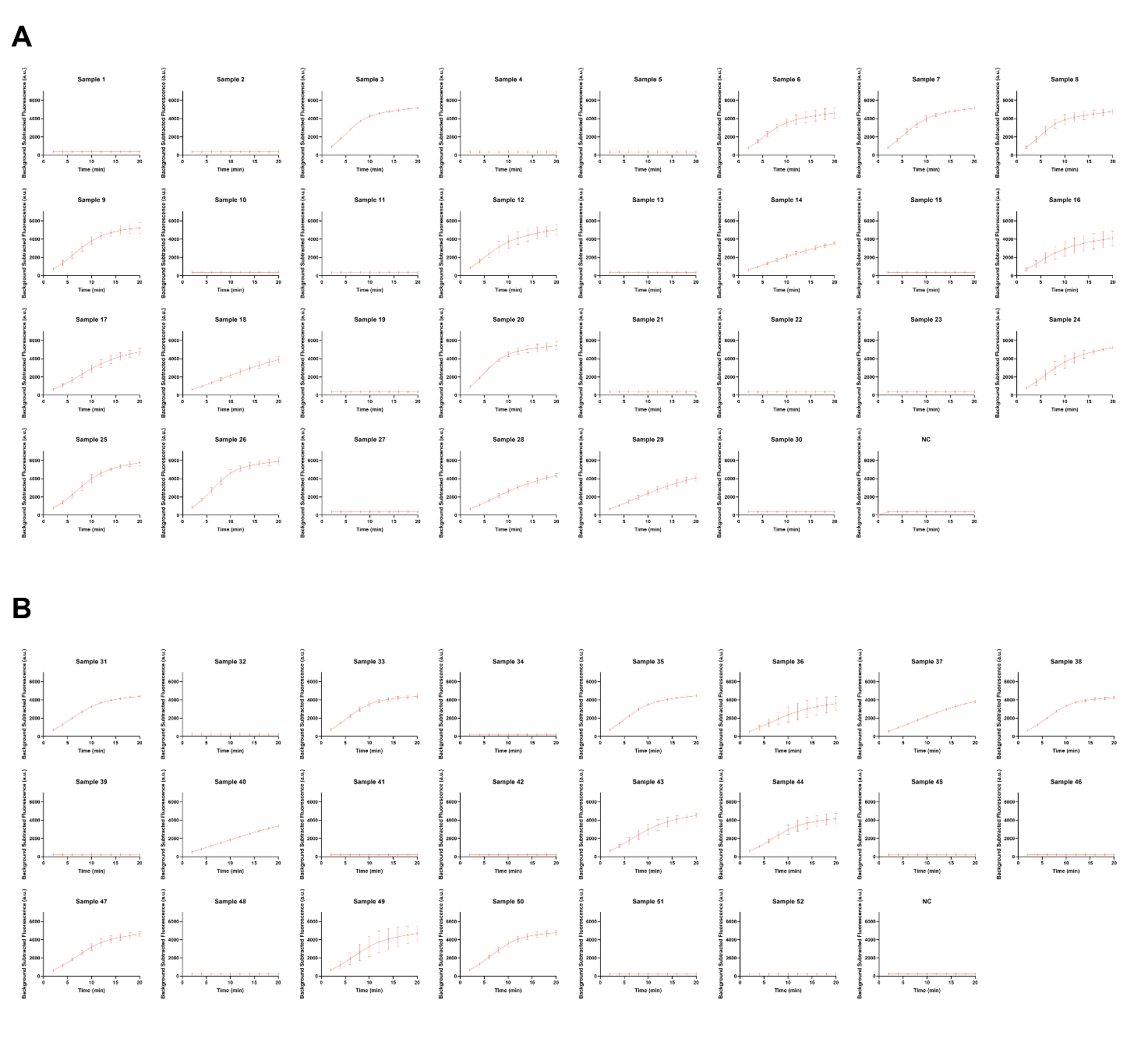


**Supplementary Figure 4.** The fluorescent kinetic curves of 28 SFTSV-infected patient samples and 24 healthy control samples using Cas12-M-crRNA8. The 28 positive patient samples resulted in robust fluorescence curves indicating the presence of the SFTSV under the standard amplification and detection conditions (20 min amplification and signal within 20 min). NC, no-template control (n=52, three replicates).

**
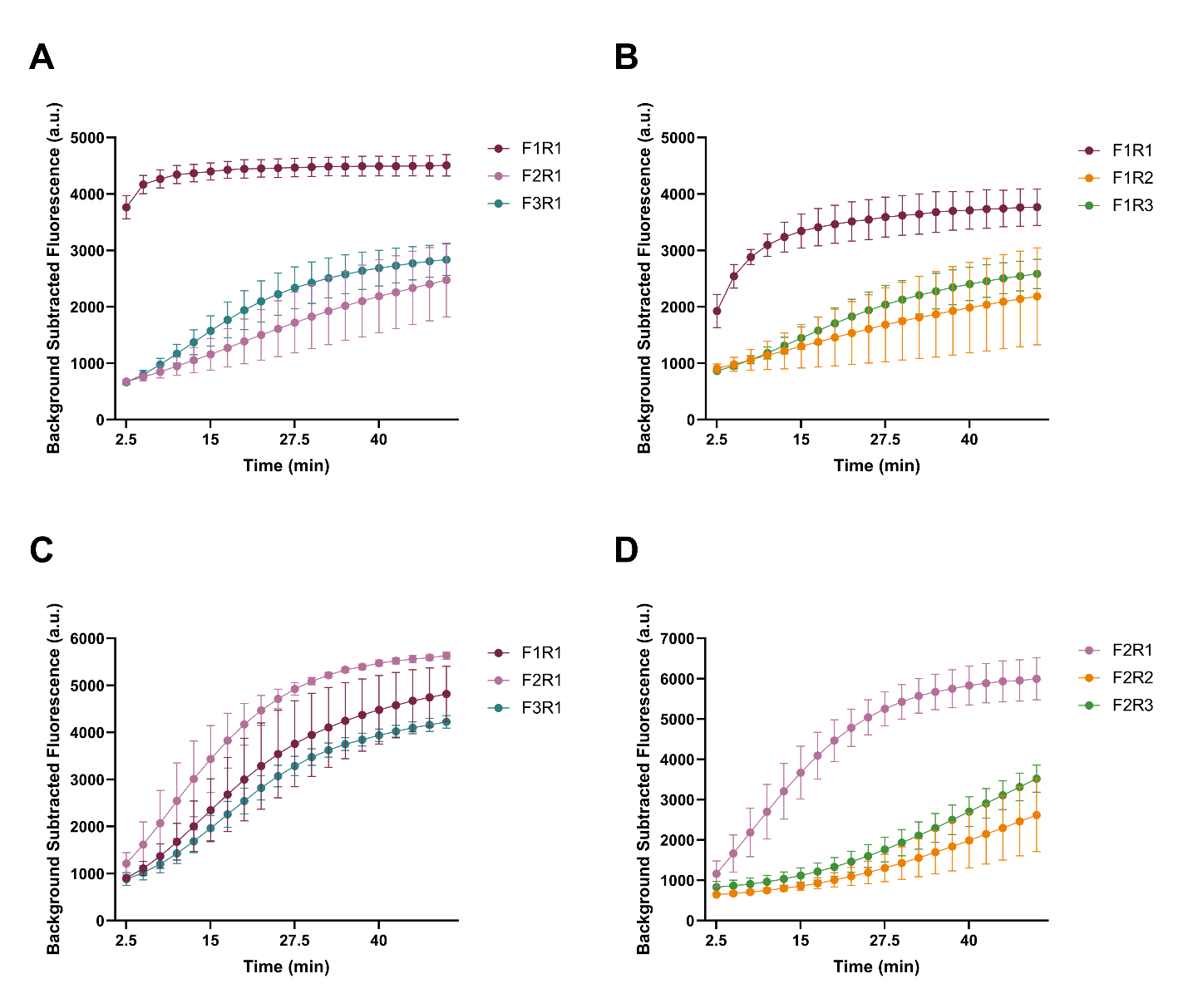
**

**Supplementary Figure 5.** Primer Screening. Primers of Cas13-L-crRNA1 (A, B) and Cas13-L-crRNA2 (C, D) were screened using a single reverse primer against the corresponding forward primers, the forward primer with the best performance was selected to perform the second round of screening. Values represent mean±SD (n=3).

**
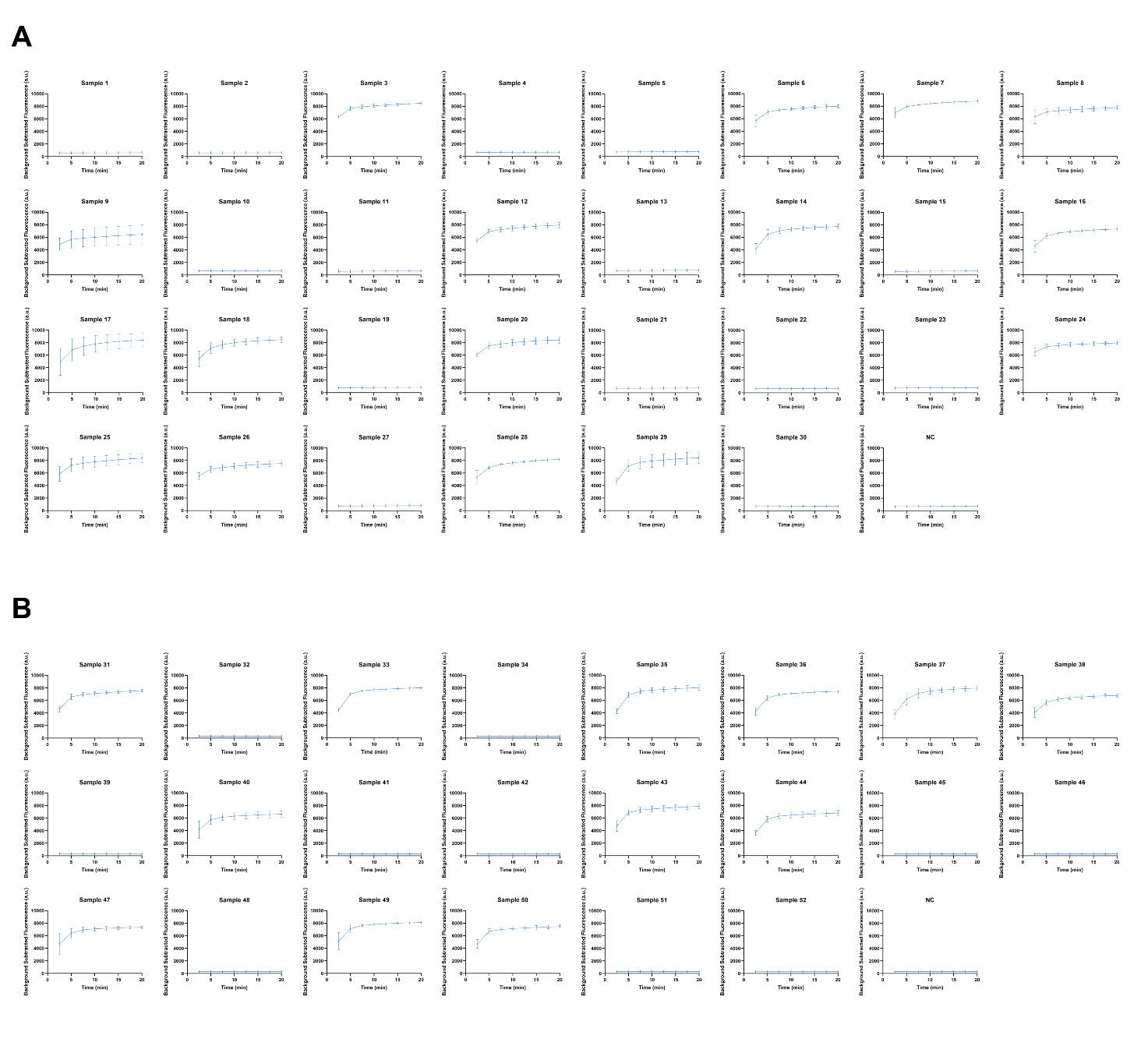
**

**Supplementary Figure 6.** The fluorescent kinetic curves on 28 SFTSV-infected patient samples and 24 healthy control samples using Cas13-L-crRNA1. The 28 positive patient samples resulted in robust fluorescence curves indicating the presence of the SFTSV under the standard amplification and detection conditions (20 min amplification and signal within 20 min). NC, no-template control (n=52, three replicates).

**
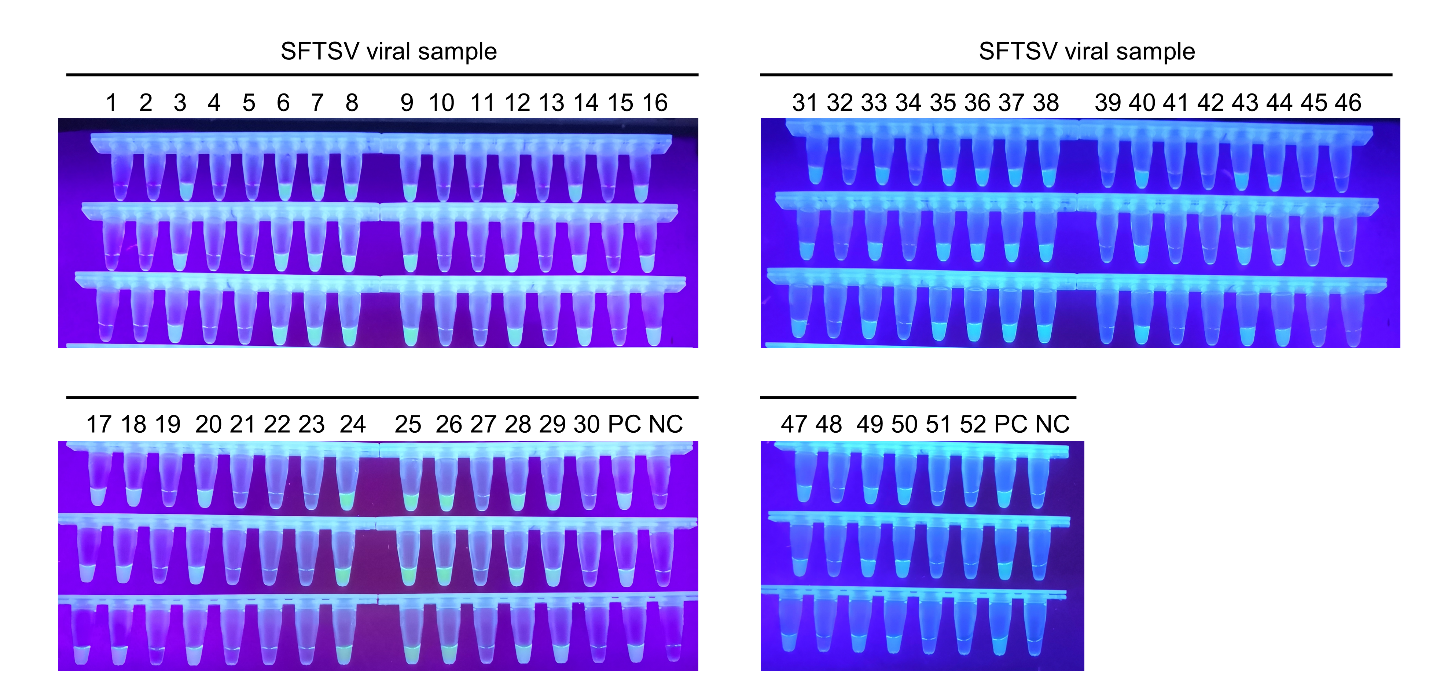
**

**Supplementary Figure 7.** The fluorescence visualization of 52 clinical samples (28 positive cases and 24 negative cases) based on CRISPR/Cas12 system using Cas12-M-crRNA8. The detection results were observed under ultraviolet light (n=3). PC, positive control; NC, no-template control.


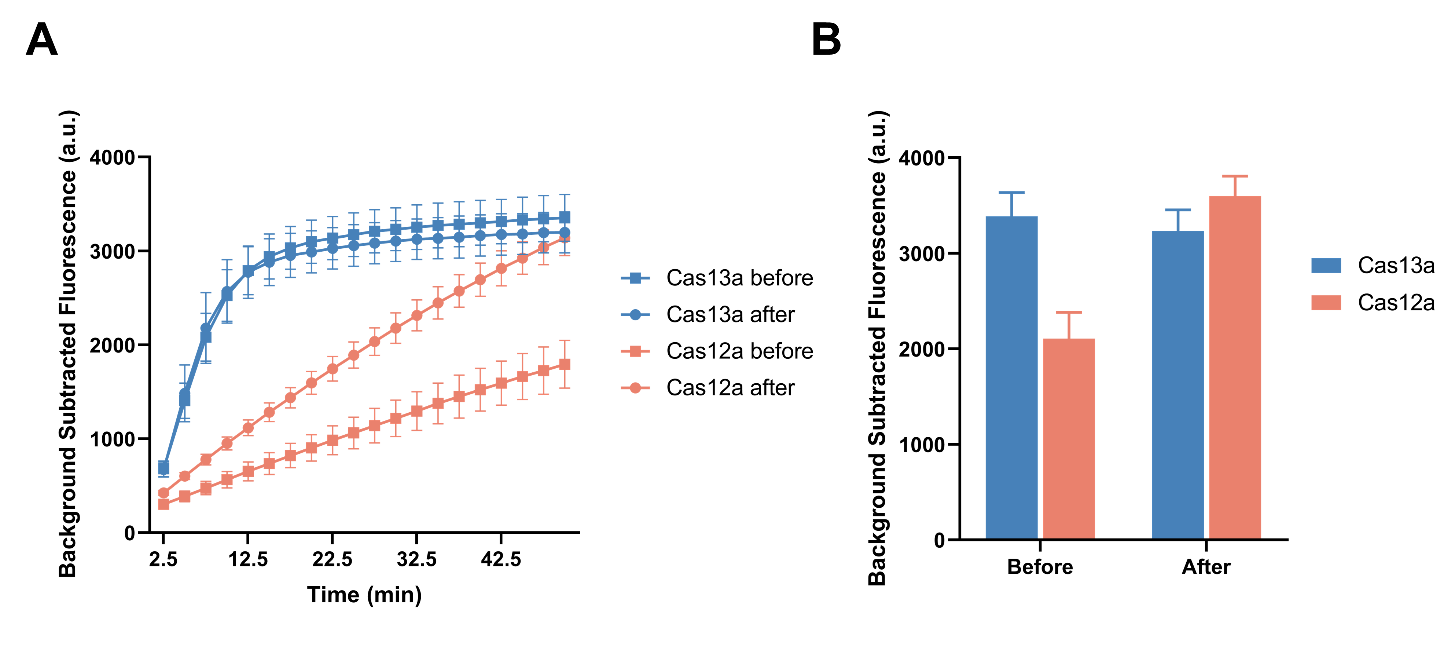


**Supplementary Figure 8.** The efficiency of Cas12a before and after the optimization of condition. Comparison the detection efficiency of Cas12 channel in before and after the optimization of condition using the synthetic SFTSV RNA standards as detection targets. Before, the all components of multiplex detection system except the two reporters was incubated together, and the concentration of rNTP was 2.5mM. After, the Cas12a protein and Cas12-crRNA was pre-incubated at 37℃ for 10 min, and the concentration of rNTP was 1mM.


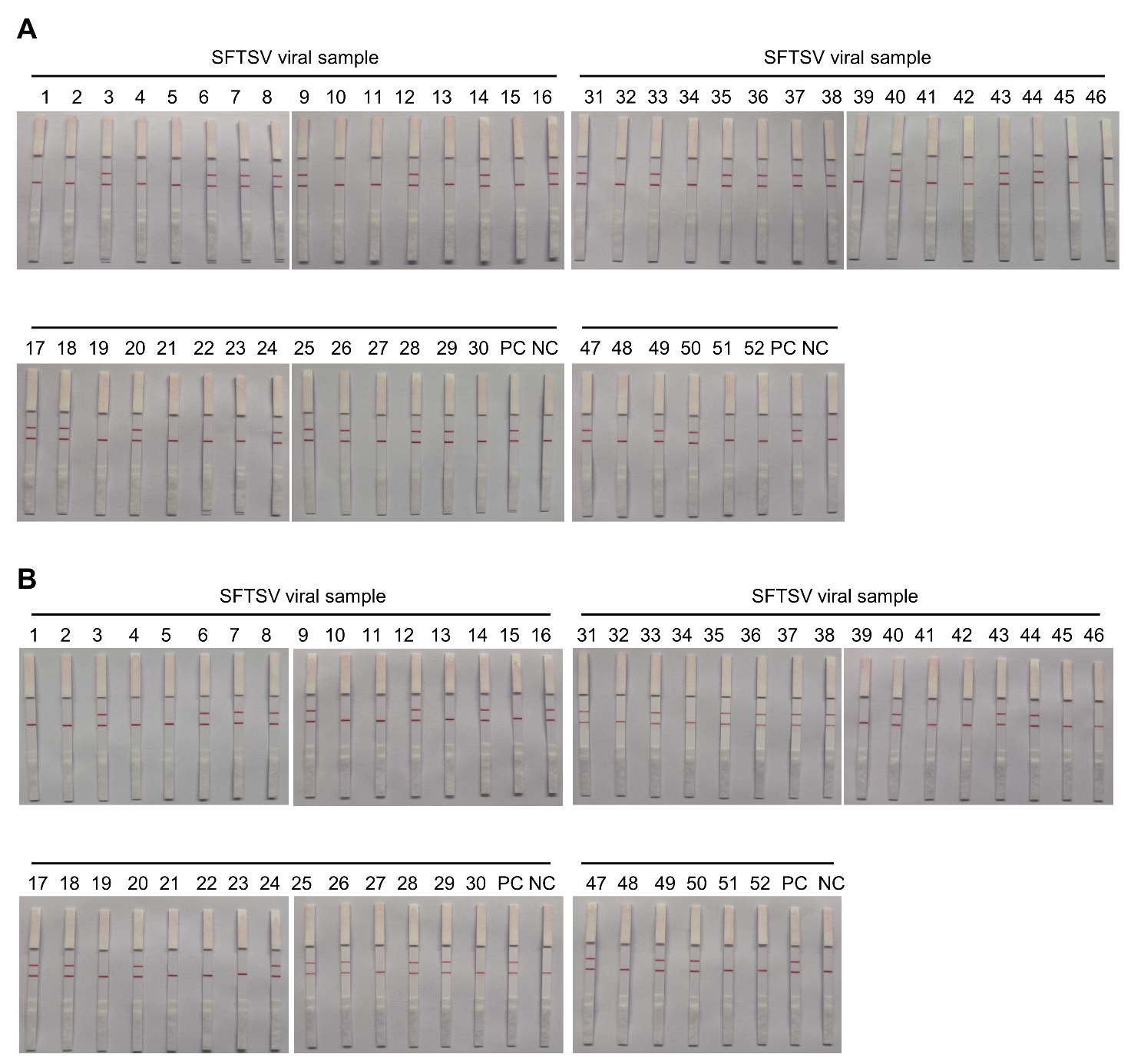


**Supplementary Figure 9.** The detection results of the 52 clinical samples (28 positive cases and 24 negative cases) with the single-target test strips. The lateral flow detection based on CRISPR/Cas12a system using Cas12-M-crRNA8 (A) and based on CRISPR/Cas13a system using Cas13-L-crRNA1. PC, positive control. NC, no-template control.
